# Supplementary material for: A Sparse-Modeling Based Approach for Class Specific Feature Selection
Source: PeerJ Comput Sci. 2019 Nov 18;5:e237. doi: 10.7717/peerj-cs.237 (PMC7924712; doi:10.7717/peerj-cs.237)
Supplement: Supplemental Information 1 [file peerj-cs-05-237-s001.pdf]

A Sparse-Modeling Based Approach for Class-Specific Feature Selection

Davide Nardone[1,*], **Angelo Ciaramella**[1], **and Antonino Staiano**[1]

[1] *Dipartimento di Scienze e Tecnologie, Università degli Studi di Napoli "Parthenope", Centro Direzionale, Isola C4, 80143, Naples, Italy*
[*] *Corresponding author: davide.nardone@studenti.uniparthenope.it*

1. INTRODUCTION

As previously stated in the paper, SMBA-CSFS performs better when a dataset is made of many classes and when a low number of features are selected (up to 80). In order to obtain statistical support for this claim, we have applied Non-Parametric multiple comparison test (all vs all) which is a default two-steps procedure used to compare more than two groups of samples (algorithms):

- A rank-based test used for comparing and assigning ranks to each algorithm
- A post-hoc test used to find where the differences occurred between groups (algorithms), by providing the corresponding p-values

In this work, we adopted a *Friedman test* to rank the algorithms' performance across multiple datasets, taking into account the accuracy results achieved on subsets of 20 and 80 features by using the SVM classifier. This involves ranking each row together and then considering the values of ranks by columns where the best performing algorithm gets the rank of 1, the second best rank 2 and so on. We also have reported the *Cumulative Rank* (CR) considering either all the dataset or a subset of them ($CR_{\geq 5}$). Finally, a *Nemenyi post-hoc test (all vs all)* is ran to compare all the other methods against SMBA-CSFS with the aim to determine which groups are significantly different, with respect to SMBA-CSFS. The significance level α is set to 5%.

Tables S1 and S3, provide the ranking of the classification procedures based on TFS against SMBA-CSFS, for the top 20 and 80 features, respectively. Tables S2 and S4 show the p-values corresponding to Table S1 and S3.
Tables S5 and S7, provide the ranking of the classification procedures based on GF-CSFS against SMBA-CSFS, for the top 20 and 80 features, respectively. Tables S6 and S8, show the p-values corresponding to Table S5 and S7.
Table S9 provides the averaged Elapsed CPU Time (seconds) for each feature selection methods used. Note that, computational time shown here account for a single CV run, where the steps of feature selection and classification are performed. Additional results on other classifiers can be found in the supplementary material.

Table S1. **Friedman ranking values for the top 20 accuracy results among TFS and SMBA-CSFS. Lower values indicates better performance. The different colours represent the first three rank places: red for the first place, blue for the second place and green for the third place.**

	ALLAML(2)	LEUKEMIA(2)	CLL_SUB_111(3)	GLIOMA(4)	LUNG_C(5)	LUNG_D(7)	DLBCL(9)	CAR(11)	GCM(14)	*CR*	$CR_{\geq 5}$
Fisher	2.13	2.6	4.2	**2.68**	8.95	9.18	8.15	10.6	8.95	*57.44*	*45.83*
Relief	3.45	3.33	2.4	3.28	5.78	2.28	3.85	3.53	7.88	***35.78***	***23.32***
mRmR	10.4	**1.6**	7.05	8.3	8.8	4	4.45	4.48	2.25	*51.33*	***23.98***
MI	**2.05**	2.475	**2.3**	3.05	4.9	6.03	4.95	1.9	7.70	***35.36***	*25.48*
ls-21	9.325	10.875	10.85	10.78	10.6	7.23	9.55	10.1	7.52	*86.83*	*45.00*
ll-21	9.775	5.9	5.73	6.25	4.55	6.18	9	6.6	5.55	*59.54*	*31.88*
RFS	7.925	8.4	9.95	8.98	8.83	8.73	9.95	7.7	9.25	*79.72*	*44.46*
LASSO	3.45	9	7.8	3.65	3.53	6.83	4.3	6.7	5.15	*50.41*	*26.51*
EN	3.45	9	7.8	3.65	3.53	6.83	4.3	6.7	5.15	*50.41*	*26.51*
SMBA	5.95	5.9	5.98	7.85	5.05	7.7	4.4	6.6	5.55	*54.98*	*29.30*
SMBA-CSFS	7.525	5.925	3.55	4.55	**1.53**	**1.22**	**2.16**	**1.19**	**1.05**	***28.70***	***7.15***

Table S2. **Friedman p-values for the top 20 accuracy results among TFS and SMBA-CSFS.**

	ALLAML	LEUKEMIA	CLL_SUB_111	GLIOMA	LUNG_C	LUNG_D	DLBCL	CAR	GCM
FS	1.00E-05	2.05E-03	1.00E+00	1.80E-04	0.00E+00	0.00E+00	8.00E-05	0.00E+00	2.74E-12
Relief	4.66E-02	3.29E-02	1.00E+00	2.52E-03	2.52E-03	1.00E+00	1.00E+00	1.00E+00	4.21E-09
mRmR	3.37E-01	2.00E-05	6.00E-05	1.00E+00	0.00E+00	2.70E-01	1.00E+00	7.10E-02	1.00E+00
MI	1.00E-05	1.21E-03	1.00E+00	9.80E-04	6.53E-02	1.20E-04	1.00E+00	1.00E+00	1.26E-08
ls-21	1.00E+00	9.12E-03	0.00E+00	1.16E-01	0.00E+00	0.00E+00	0.00E+00	0.00E+00	3.67E-08
ll-21	1.00E+00	1.00E+00	1.76E-02	1.00E+00	2.00E-01	6.00E-05	0.00E+00	1.00E-05	9.80E-04
RFS	1.00E+00	1.00E+00	0.00E+00	1.00E+00	0.00E+00	0.00E+00	0.00E+00	0.00E+00	2.93E-13
LASSO	5.62E-03	1.00E+00	0.00E+00	1.10E-02	1.00E+00	0.00E+00	1.00E+00	1.00E-05	5.09E-03
EN	5.62E-03	1.00E+00	0.00E+00	1.10E-02	1.00E+00	0.00E+00	1.00E+00	0.00E+00	5.09E-03
SMBA	1.00E+00	1.00E+00	6.83E-03	1.00E+00	3.92E-02	0.00E+00	1.00E+00	1.00E-05	9.80E-04

Table S3. **Friedman ranking values for the top 80 accuracy results among TFS and SMBA-CSFS. Lower values indicates better performance. The different colours represent the first three rank places: red for the first place, blue for the second place and green for the third place.**

	ALLAML(2)	LEUKEMIA(2)	CLL_SUB_111(3)	GLIOMA(4)	LUNG_C(5)	LUNG_D(7)	DLBCL(9)	CAR(11)	GCM(14)	*CR*	$CR_{\geq 5}$
FS	**1.88**	2.91	5.33	6.4	8.96	9.41	4.33	10.23	10.47	*59.92*	*43.4*
Relief	2.73	3.26	5.61	4.46	4.16	3.23	3.14	3.53	8.35	***38.47***	***22.41***
mRmR	10.51	**1.8**	6.93	9.2	7.23	4.28	3.3	5.08	2.11	*50.44*	***22***
MI	2.63	2.06	**2.33**	7.63	4.29	4.99	5.63	2.06	9.32	***40.94***	*26.29*
ls-21	10.18	10.97	10.73	10.39	10.44	4.41	10.58	10.7	7.61	*86.01*	*43.74*
ll-21	8.23	6.63	6.14	4.38	4.23	6.58	9.01	6.23	4.4	*55.83*	*30.45*
RFS	6.69	6.63	5.23	7.31	10.04	4.99	9.163	5.34	5.22	*60.61*	*34.75*
LASSO	4.51	9.2	6.52	3.03	4.99	7.49	5.33	7.76	6.41	*55.24*	*31.98*
EN	4.51	9.2	6.52	3.03	4.99	7.49	5.33	7.76	6.41	*55.24*	*31.98*
SMBA	6.24	6.62	8.03	4.53	2.98	9.37	3.42	6.23	4.4	*51.82*	*26.4*
SMBA-CSFS	7.89	6.73	4.66	**2.84**	**2.68**	**2.16**	**1.74**	**1.07**	**1.31**	***31.08***	***8.96***

Table S4. **Friedman p-values for the top 80 accuracy results among TFS and SMBA-CSFS.**

	ALLAML	LEUKEMIA	CLL_SUB_111	GLIOMA	LUNG_C	LUNG_D	DLBCL	CAR	GCM
FS	0.00E+00	0.00E+00	2.00E-05	1.00E+00	0.00E+00	0.00E+00	0.00E+00	0.00E+00	0.00E+00
Relief	0.00E+00	0.00E+00	0.00E+00	1.00E+00	1.00E+00	1.00E+00	0.00E+00	1.50E-04	0.00E+00
mRmR	3.00E-05	0.00E+00	0.00E+00	0.00E+00	0.00E+00	1.00E+00	0.00E+00	0.00E+00	1.00E+00
MI	0.00E+00	0.00E+00	1.00E+00	8.29E-03	1.00E+00	1.00E+00	1.25E-01	1.00E+00	0.00E+00
ls-21	7.10E-04	0.00E+00	0.00E+00	0.00E+00	0.00E+00	1.00E+00	0.00E+00	0.00E+00	0.00E+00
ll-21	1.85E-01	1.00E+00	0.00E+00	8.83E-01	1.00E+00	0.00E+00	3.44E-02	0.00E+00	2.00E-07
RFS	1.00E+00	1.00E+00	5.00E-05	8.04E-02	0.00E+00	1.00E+00	1.21E-02	0.00E+00	4.73E-12
LASSO	0.00E+00	1.30E-04	0.00E+00	3.00E-05	6.78E-01	0.00E+00	1.68E-02	0.00E+00	0.00E+00
EN	0.00E+00	1.30E-04	0.00E+00	3.00E-05	6.78E-01	0.00E+00	1.68E-02	0.00E+00	0.00E+00
SMBA	9.47E-02	1.00E+00	0.00E+00	1.00E+00	1.00E+00	0.00E+00	0.00E+00	0.00E+00	2.00E-07

Table S5. **Friedman ranking values for the top 20 accuracy results among GF-CSFS and SMBA-CSFS. Lower values indicates better performance. The different colours represent the first three rank places: red for the first place, blue for the second place and green for the third place.**

	ALLAML(2)	LEUKEMIA(2)	CLL_SUB_111(3)	GLIOMA(4)	LUNG_C(5)	LUNG_D(7)	DLBCL(9)	CAR(11)	GCM(14)	*CR*	$CR_{\geq 5}$
FS	3	**1.88**	2.55	**1.43**	4.05	4.25	3.95	2.5	2.08	***25.69***	***16.83***
Relief	4.83	3.63	**1.5**	3.5	6.35	4.73	4.08	4.8	3.88	*37.3*	*23.84*
mRmR	9.1	1.88	6.85	7.8	8.2	4.78	5.33	7.6	4.47	*56.01*	*30.38*
MI	2.58	2.25	2.25	2.8	3.13	6.2	3.08	2.73	3.32	***28.34***	***18.46***
ls-21	8.1	9	9.53	9.2	9.78	9.45	10	9.95	6.05	*81.06*	*45.23*
ll-21	8.3	6.35	5.48	6.68	6.68	8.4	8.3	7.65	8.05	*65.89*	*39.08*
RFS	7.23	6.4	8.1	8.45	8.98	8.75	8.6	8.8	8.4	*73.71*	*43.53*
LASSO	**2.3**	8.5	7.9	4.48	3.08	3.7	3.2	4.65	8.65	*46.46*	*23.28*
EN	2.45	8.5	7.15	4.48	3.73	3.7	2.7	4.85	8.65	*46.21*	*23.63*
SMBA-CSFS	6.15	5.25	3.7	5.2	**1.05**	**1.05**	**2.18**	**2.25**	**1.45**	***28.28***	***7.98***

Table S6. **Friedman p-values for the top 20 accuracy results among GF-CSFS and SMBA-CSFS.**

	ALLAML	LEUKEMIA	CLL_SUB_111	GLIOMA	LUNG_C	LUNG_D	DLBCL	CAR	GCM
FS	1.00E-05	2.05E-03	1.00E+00	1.80E-04	0.00E+00	0.00E+00	8.00E-05	0.00E+00	1.00E+00
Relief	4.66E-02	3.29E-02	1.00E+00	2.52E-03	2.52E-03	1.00E+00	1.00E+00	1.00E+00	5.09E-01
mRmR	3.37E-01	2.00E-05	6.00E-05	1.00E+00	0.00E+00	2.70E-01	1.00E+00	7.10E-02	7.11E-02
MI	1.00E-05	1.21E-03	1.00E+00	9.80E-04	6.53E-02	1.20E-04	1.00E+00	1.00E+00	1.00E+00
ls-21	1.00E+00	9.12E-03	0.00E+00	1.16E-01	0.00E+00	0.00E+00	0.00E+00	0.00E+00	6.98E-05
ll-21	1.00E+00	1.00E+00	1.76E-02	1.00E+00	2.00E-01	6.00E-05	0.00E+00	1.00E-05	2.45E-10
RFS	1.00E+00	1.00E+00	0.00E+00	1.00E+00	0.00E+00	0.00E+00	0.00E+00	0.00E+00	1.75E-11
LASSO	5.62E-03	1.00E+00	0.00E+00	1.10E-02	1.00E+00	0.00E+00	1.00E+00	1.00E-05	2.46E-12
EN	5.62E-03	1.00E+00	0.00E+00	1.10E-02	1.00E+00	0.00E+00	1.00E+00	0.00E+00	2.46E-12

Table S7. Friedman ranking values for the top 80 accuracy results among GF-CSFS and SMBA-CSFS. Lower values indicates better performance. The different colours represent the first three rank places: red for the first place, blue for the second place and green for the third place.

	ALLAML(2)	LEUKEMIA(2)	CLL_SUB_111(3)	GLIOMA(4)	LUNG_C(5)	LUNG_D(7)	DLBCL(9)	CAR(11)	GCM(14)	*CR*	$CR_{\geq 5}$
FS	**2.59**	2.58	3.61	3.94	4.44	5.64	3.91	2.56	2.72	***31.99***	***19.27***
Relief	3.78	3.19	2.23	4.98	6.59	5.25	**2.53**	3.74	4.84	*37.13*	*22.95*
mRmR	9.61	**2.04**	7.76	8.69	8.52	5.15	4.6	7.18	4.3	*57.85*	*29.75*
MI	4.1	2.7	**2.09**	3.75	4.09	6.93	4.29	3.65	3.41	***35.01***	***22.37***
ls-21	8.91	9.75	9.66	9.65	9.89	8.37	9.95	9.99	3.41	*79.58*	*41.61*
ll-21	7.58	8.09	4.53	7.73	6.56	9.16	8.2	8.11	3.41	*63.37*	*35.44*
RFS	6.03	6.58	5.19	7.15	8.41	7.89	7.41	8.72	3.41	*60.79*	*35.84*
LASSO	2.94	7.02	7.66	**2.44**	2.36	2.79	2.98	4.98	9.29	*42.46*	*22.4*
EN	3.09	7.02	7.64	**2.44**	2.56	2.79	3.1	4.88	9.29	*42.81*	*22.62*
SMBA-CSFS	6.38	6.04	4.13	5.23	**1.57**	**1.53**	3.54	**1.19**	**1.47**	***31.08***	***9.3***

Table S8. Friedman p-values for the top 80 accuracy results among GF-CSFS and SMBA-CSFS.

	ALLAML	LEUKEMIA	CLL_SUB_111	GLIOMA	LUNG_C	LUNG_D	DLBCL	CAR	GCM
FS	0.00E+00	0.00E+00	2.00E-05	1.00E+00	0.00E+00	0.00E+00	0.00E+00	0.00E+00	3.91E-01
Relief	0.00E+00	0.00E+00	0.00E+00	1.00E+00	1.00E+00	1.00E+00	0.00E+00	1.50E-04	8.83E-11
mRmR	3.00E-05	0.00E+00	0.00E+00	0.00E+00	0.00E+00	1.00E+00	0.00E+00	0.00E+00	1.50E-07
MI	0.00E+00	0.00E+00	1.00E+00	8.29E-03	1.00E+00	1.00E+00	1.25E-01	1.00E+00	2.20E-03
ls-21	7.10E-04	0.00E+00	0.00E+00	0.00E+00	0.00E+00	1.00E+00	0.00E+00	0.00E+00	0.00E+00
ll-21	1.85E-01	1.00E+00	0.00E+00	8.83E-01	1.00E+00	0.00E+00	3.44E-02	0.00E+00	0.00E+00
RFS	1.00E+00	1.00E+00	5.00E-05	8.04E-02	0.00E+00	1.00E+00	1.21E-02	0.00E+00	2.34E-11
LASSO	0.00E+00	1.30E-04	0.00E+00	3.00E-05	6.78E-01	0.00E+00	1.68E-02	0.00E+00	0.00E+00
EN	0.00E+00	1.30E-04	0.00E+00	3.00E-05	6.78E-01	0.00E+00	1.68E-02	0.00E+00	0.00E+00

Table S9. Elapsed CPU Time (seconds) computed on each dataset. The computational time accounts for a single CV run, when the feature selection and classification steps are performed.

Elapsed CPU Time (seconds)									
	ALLAML	LEUKEMIA	CLL_SUB_111	GLIOMA	LUNG_C	LUNG_D	DLBCL	CAR	GCM
FS	0.022	0.1	0.06	0.04	0.15	0.07	1.35	0.22	0.28
Relief	0.046	0.08	0.12	0.04	0.35	0.07	0.18	0.22	3.19
mRmR	1,699.76	1,640.54	4,269.87	1,081	5,112.4	66.99	4,156.47	7,942.55	52,634
MI	34.7	51.04	80.7	57.24	47.5	4.97	106.29	250.8	144.44
ls-21	22.22	30.42	42.39	26.96	6.15	1.61	6.03	137.17	185.11
ll-21	27.08	33.64	52.92	28.52	8.4	4.55	9.18	142.57	1.91
RFS	973.3	1,711.42	4,029.69	1,058.56	524.8	192.08	2,586.51	6,990.28	2,758.67
LASSO	2.76	2.58	4.02	1.52	8.25	0.28	4.59	7.04	0.34
EN	2.1	2.68	4.02	1.28	7.85	0.224	4.05	9.79	0.42
SMBA	10,776.88	4,372.52	20,385.78	5,790.76	1,166.6	74.97	12,393.63	36,313.31	37,786
SMBA-CSFS	11,347.19	4,811.66	22,470.86	5,833.19	1,330.02	86.4	13,125.63	39,227.57	81,103
