# Supplementary material for: A Sparse-Modeling Based Approach for Class Specific Feature Selection
Source: PeerJ Comput Sci. 2019 Nov 18;5:e237. doi: 10.7717/peerj-cs.237 (PMC7924712; doi:10.7717/peerj-cs.237)
Supplement: Supplemental Information 2 — The model evaluation metrics were computed taking into account the top 20 and 80 selected features with a 5 k-fold CV. [file peerj-cs-05-237-s002.pdf]

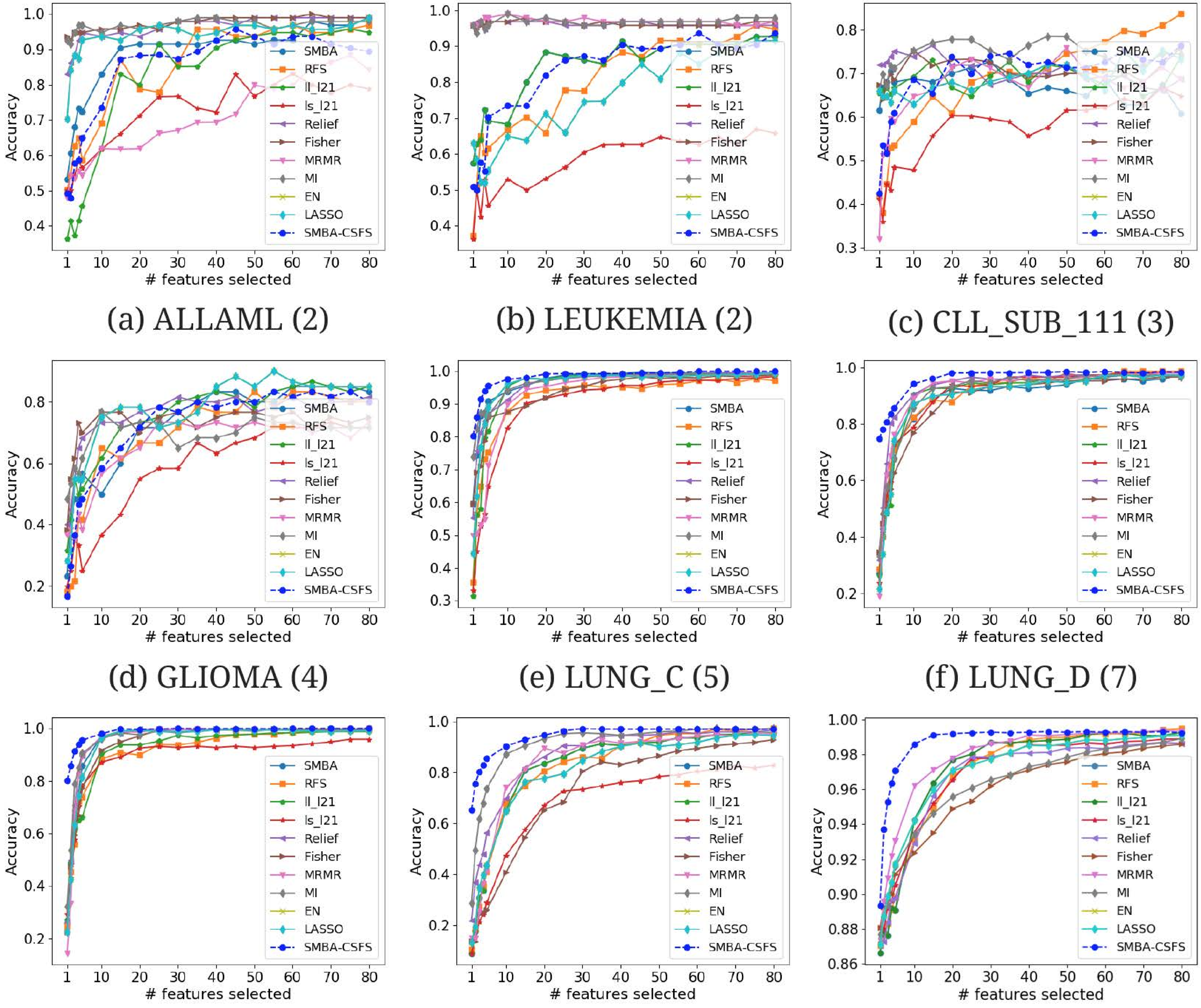

(a) ALLAML (2)
(b) LEUKEMIA (2)
(c) CLL_SUB_111 (3)
(d) GLIOMA (4)
(e) LUNG_C (5)
(f) LUNG_D (7)
(g) DLBCL (9)
(h) CARCINOM (11)
(i) GCM (14)

Figure 1. Comparison of several TFS accuracies against SMBA and SMBA-CSFS on nine data sets, when a varying number of features is selected. SVM classifier with 5-fold CV was used.

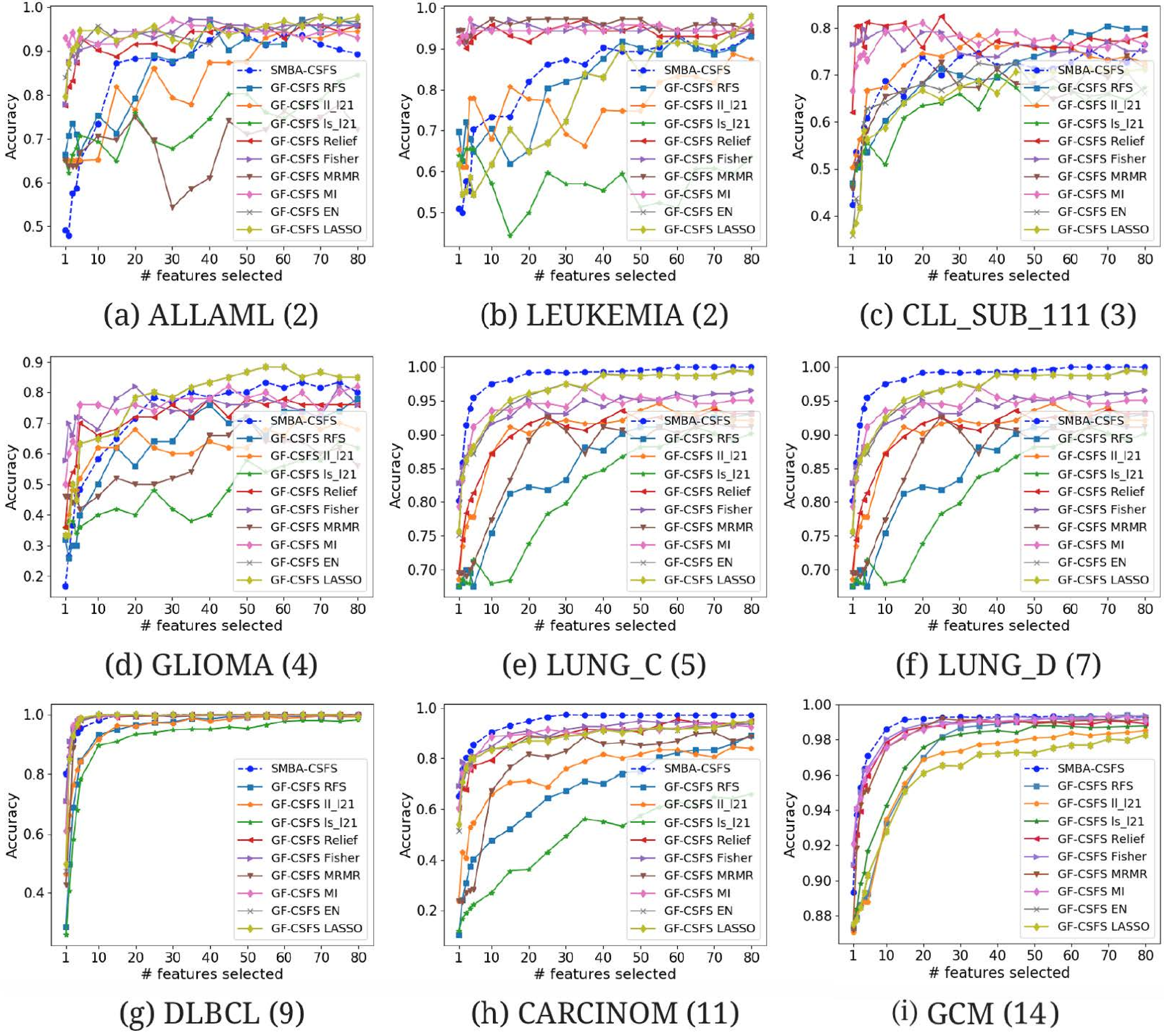

Figure 2. Comparison of several CSFS accuracies against SMBA-CSFS on nine data sets, when a varying number of features is selected. SVM classifier with 5-fold CV was used.

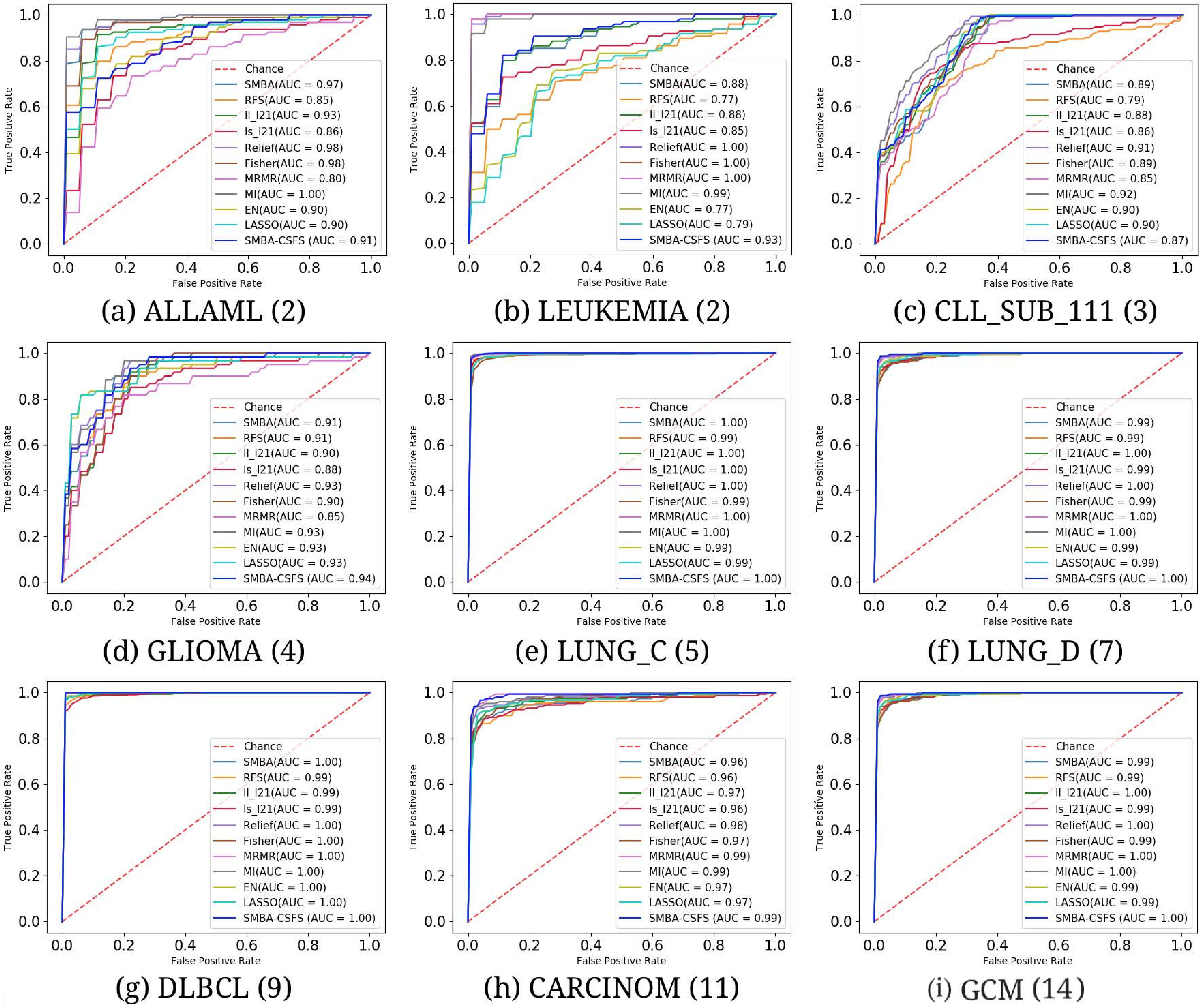

Figure 3. Averaged ROC curves comparing the performance among SMBA-CSFS and TFS methods for the classification of nine data sets on the first 20 features. SVM classifier with 5-fold CV was used.

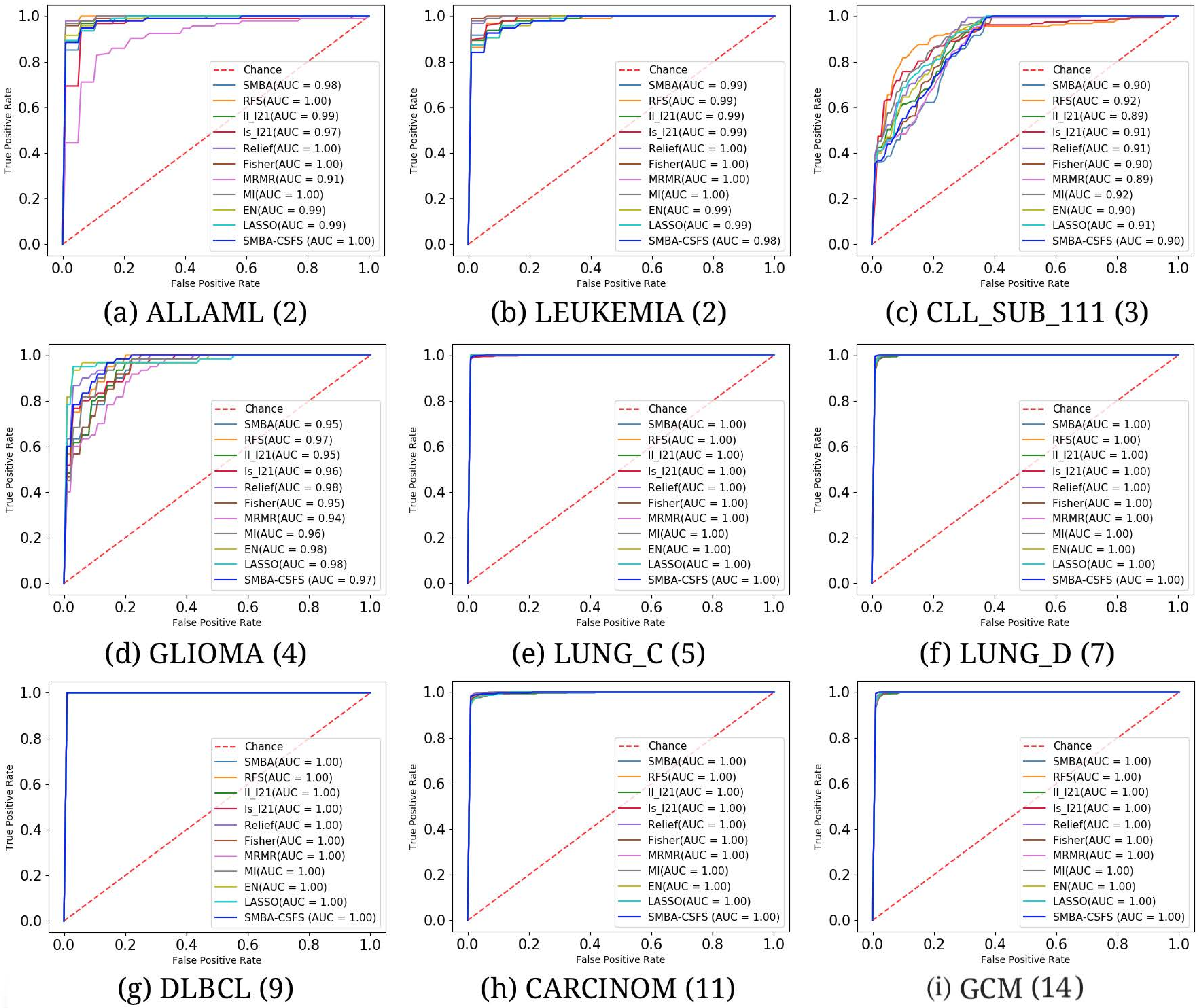

Figure 4. Averaged ROC curves comparing the performance among SMBA-CSFS and TFS methods for the classification of nine data sets on the first 80 features. SVM classifier with 5-fold CV was used.

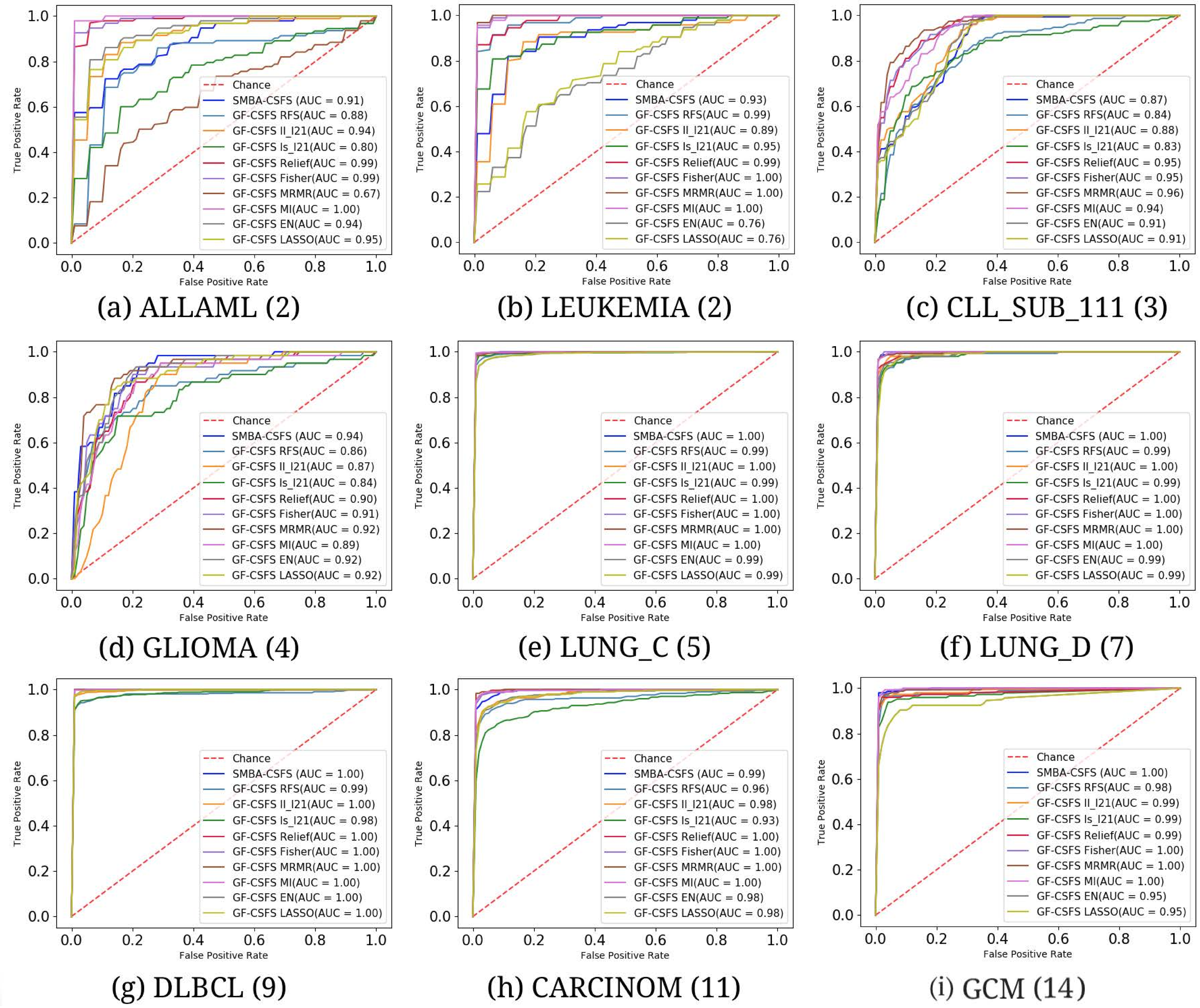

Figure 5. Averaged ROC curves comparing the performance among SMBA-CSFS and several CSFS methods for the classification of nine data sets on the first 20 features. SVM classifier with 5-fold CV was used.

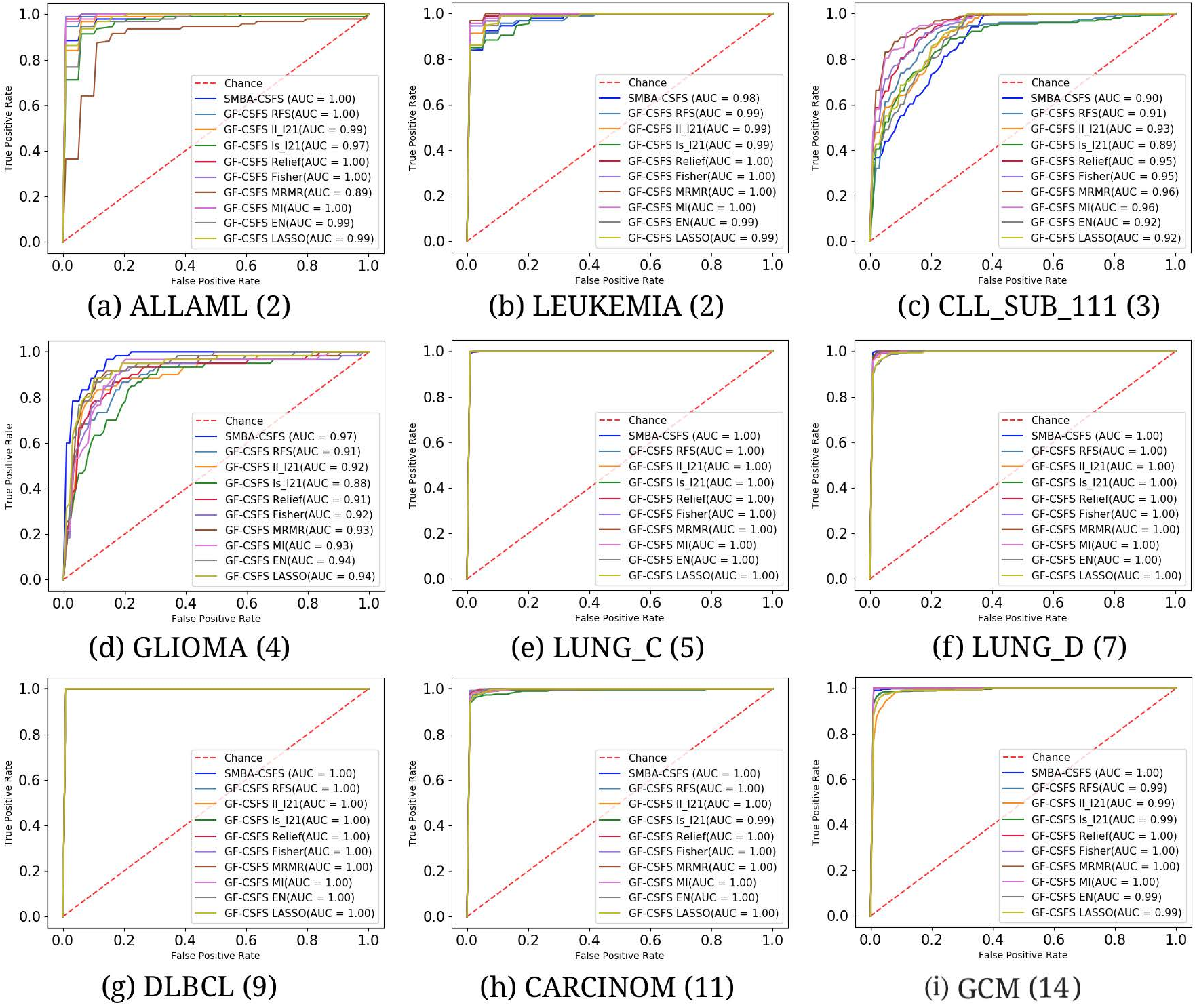

Figure 6. Averaged ROC curves comparing the performance among SMBA-CSFS and several CSFS methods for the classification of nine data sets on the first 80 features. SVM classifier with 5-fold CV was used.
