# Supplementary material for: A Sparse-Modeling Based Approach for Class Specific Feature Selection
Source: PeerJ Comput Sci. 2019 Nov 18;5:e237. doi: 10.7717/peerj-cs.237 (PMC7924712; doi:10.7717/peerj-cs.237)
Supplement: Supplemental Information 6 — Accuracies plots and ROC curves comparison between the state of art methods and the proposed methods SMBA and SMBA-CSFS on nine data sets. [file peerj-cs-05-237-s006.pdf]

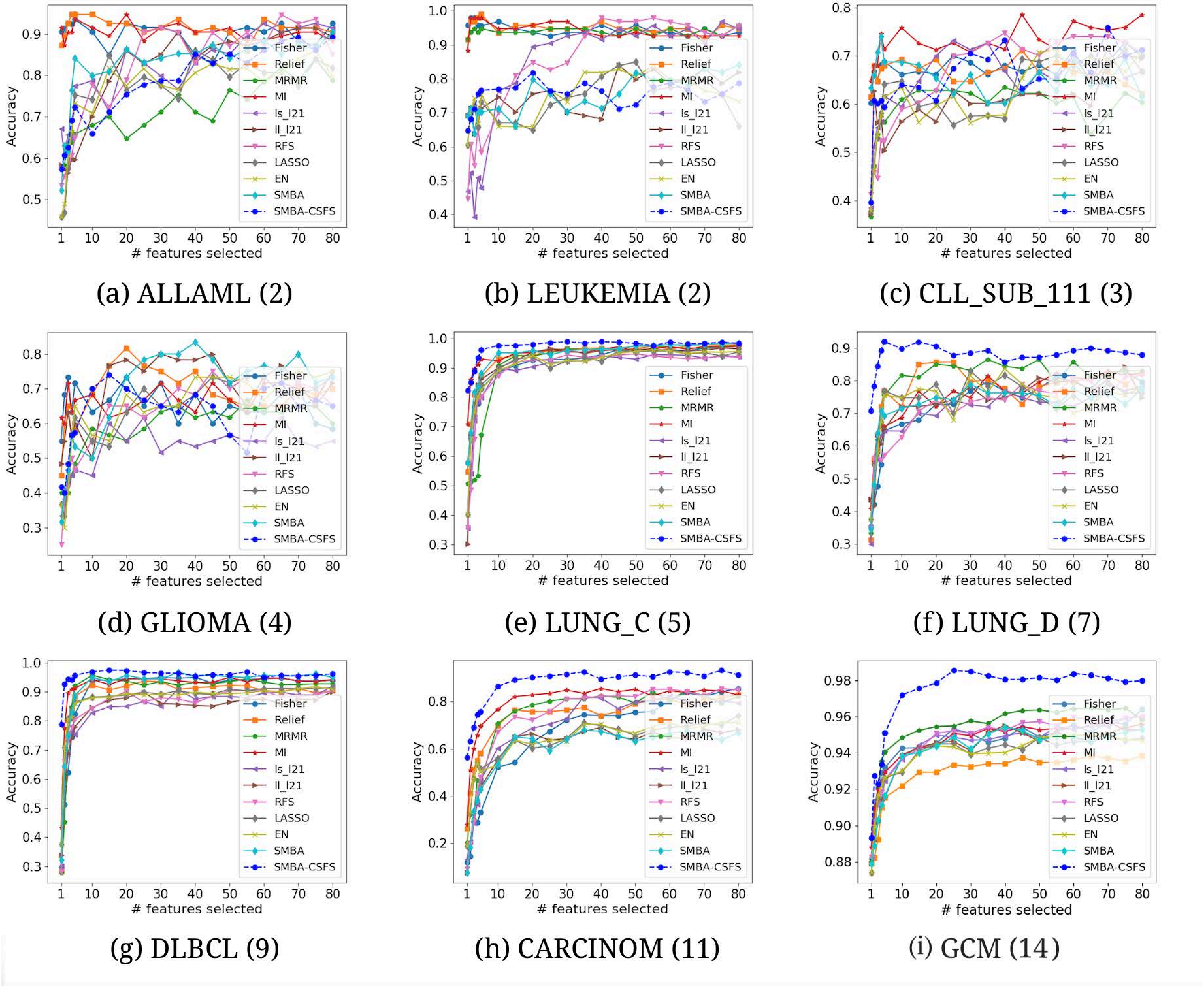

Figure 1. Comparison of several TFS accuracies against SMBA and SMBA-CSFS on nine data sets, when a varying number of features is selected. Decision Tree classifier with 5-fold CV was used.

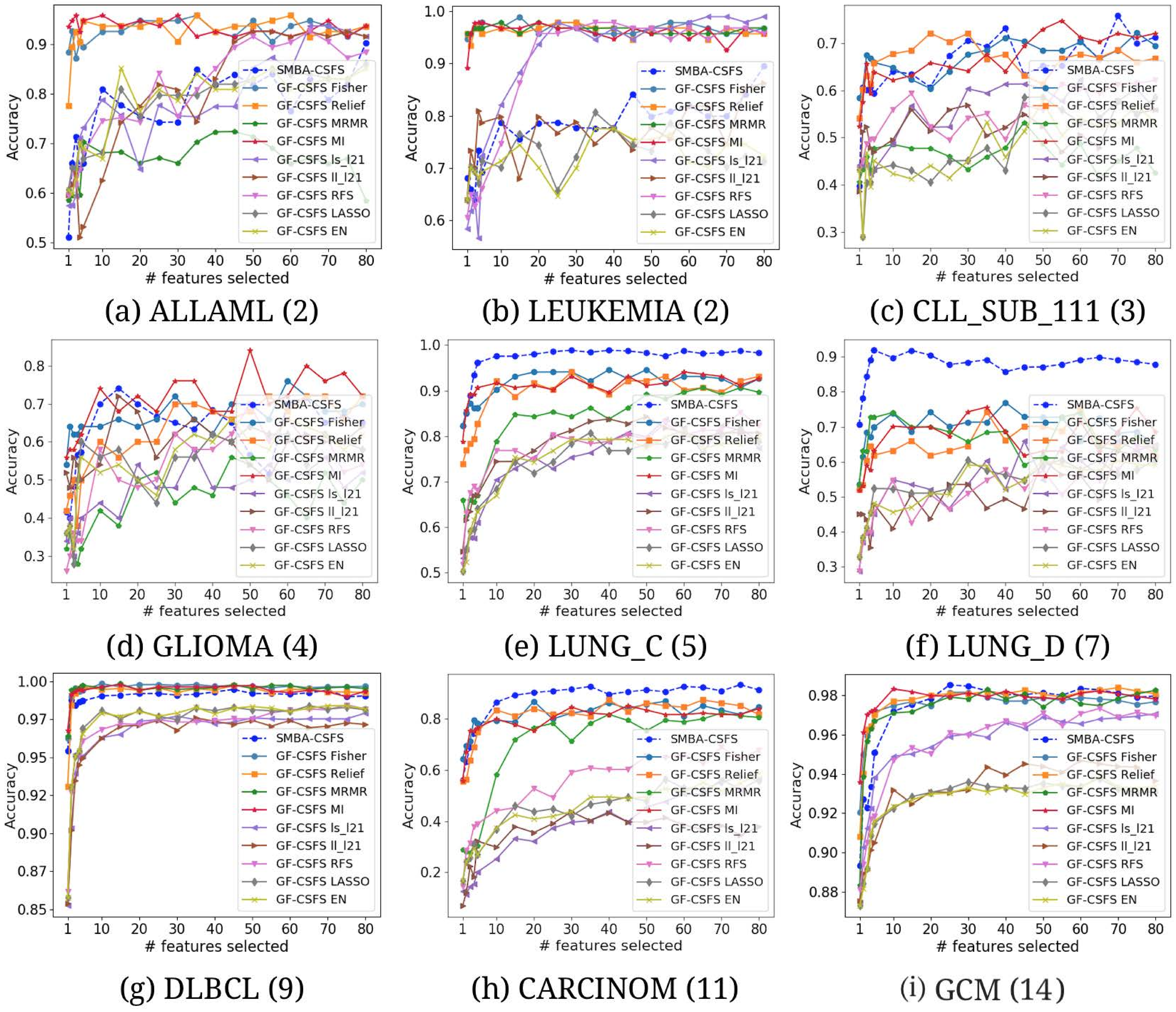

Figure 2. Comparison of several CSFS accuracies against SMBA-CSFS on nine data sets, when a varying number of features is selected. Decision Tree classifier with 5-fold CV was used.

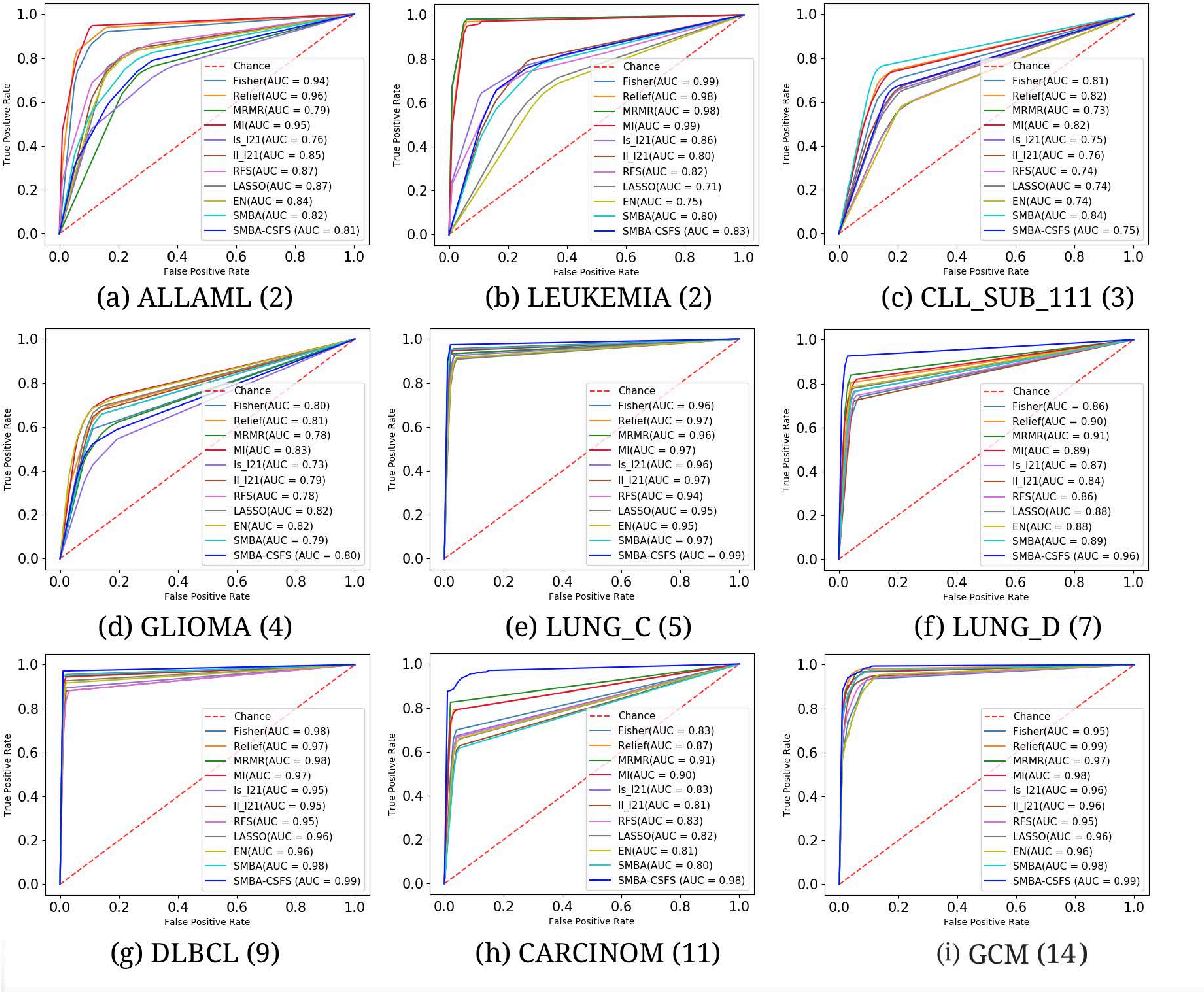

Figure 3. Averaged ROC curves comparing the performance among SMBA-CSFS and TFS methods for the classification of nine data sets on the first 20 features. Decision Tree classifier with 5-fold CV was used.

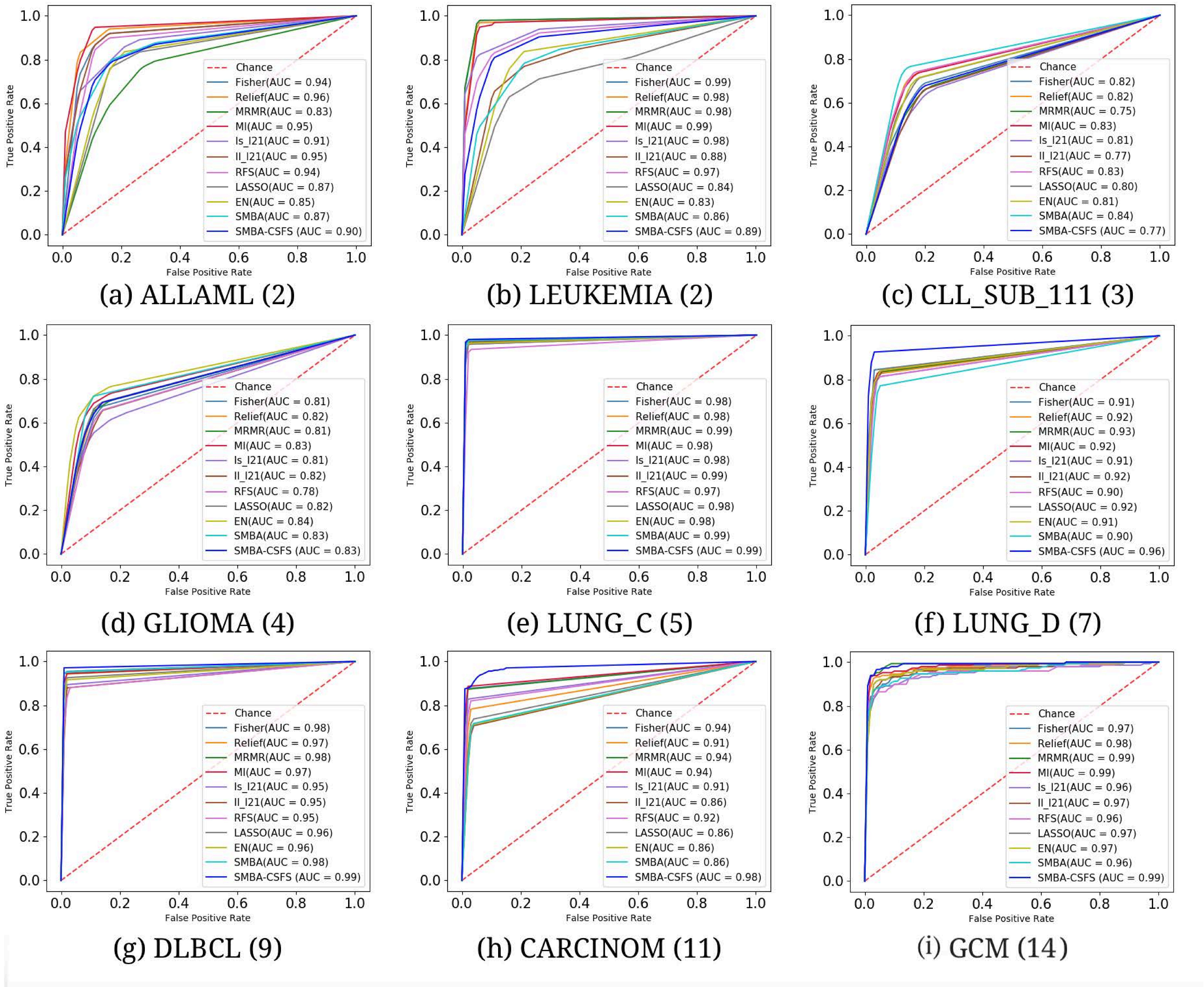

Figure 4. Averaged ROC curves comparing the performance among SMBA-CSFS and TFS methods for the classification of nine data sets on the first 80 features. Decision Tree classifier with 5-fold CV was used.

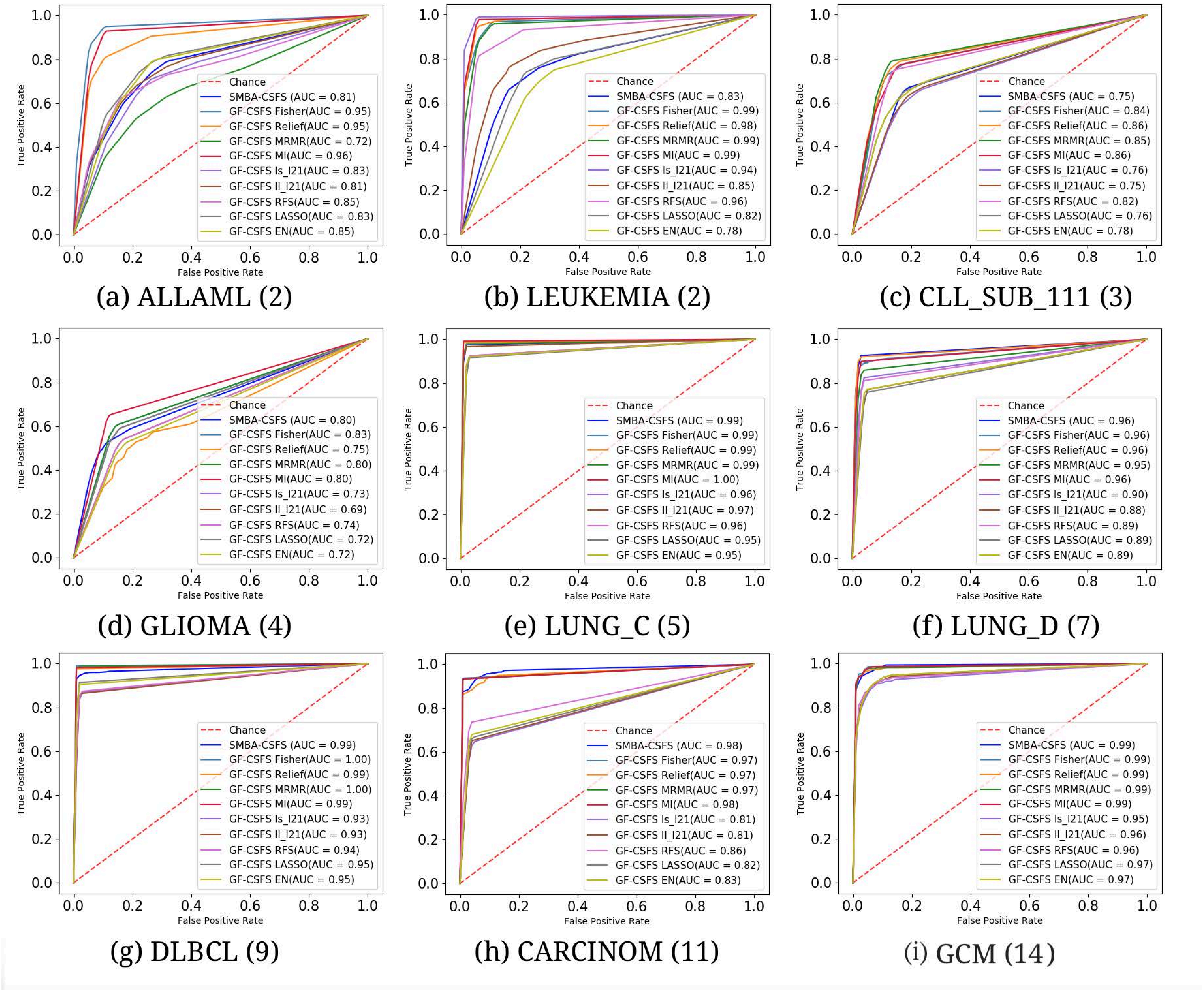

Figure 5. Averaged ROC curves comparing the performance among SMBA-CSFS and several CSFS methods for the classification of nine data sets on the first 20 features. Decision Tree classifier with 5-fold CV was used.

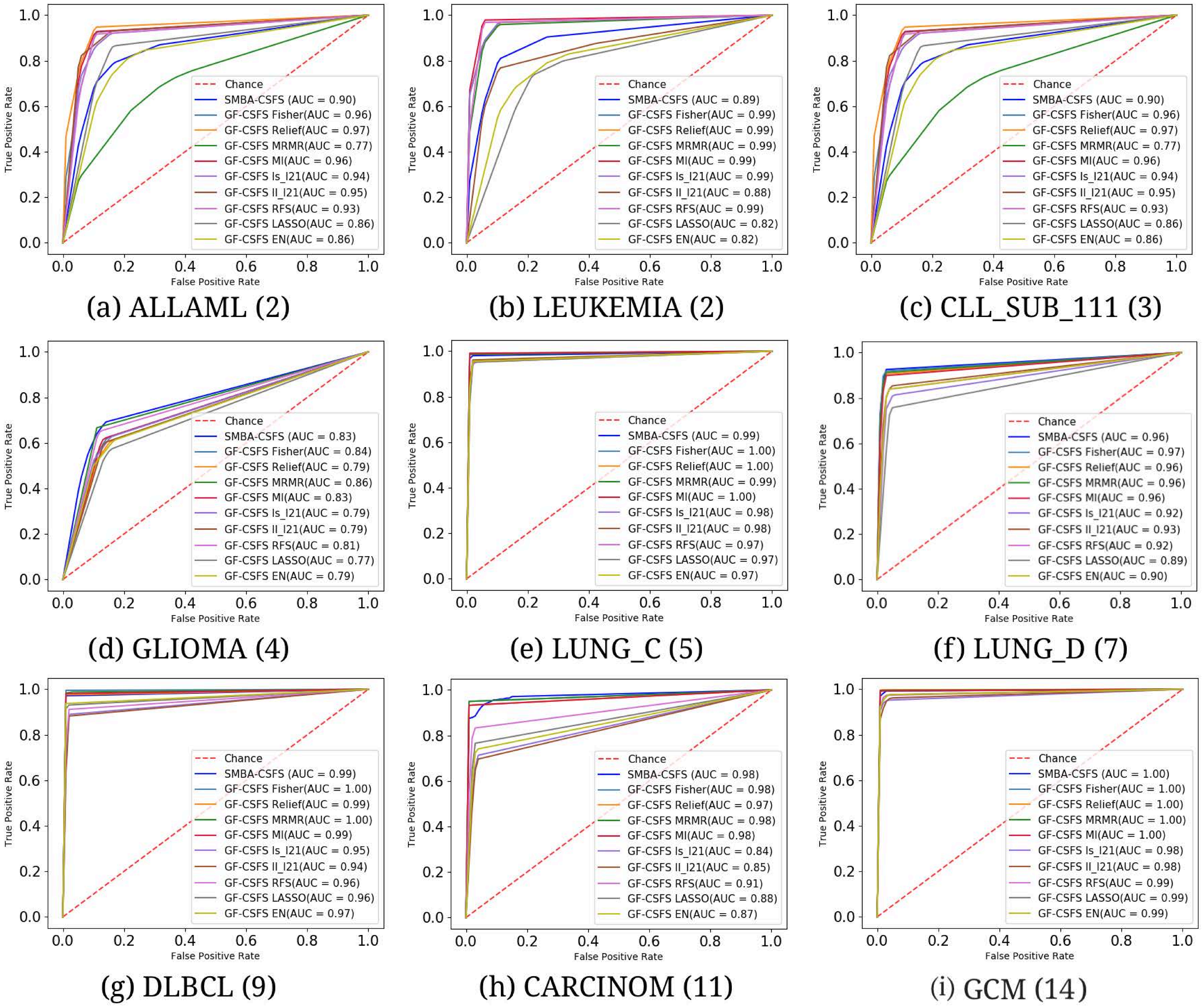

Figure 6. Averaged ROC curves comparing the performance among SMBA-CSFS and several CSFS methods for the classification of nine data sets on the first 80 features. Decision Tree classifier with 5-fold CV was used.
